# Ultrasound-Assisted Natural Deep Eutectic Solvent Extraction and Bioactivities of Flavonoids in *Ampelopsis grossedentata* Leaves

**DOI:** 10.3390/foods11050668

**Published:** 2022-02-24

**Authors:** Shiyu Zhen, Si Chen, Sheng Geng, Hao Zhang, Yongsheng Chen, Benguo Liu

**Affiliations:** 1 School of Food Science, Henan Institute of Science and Technology, Xinxiang 453003, China; jysyzhen@126.com (S.Z.); chensi0856@126.com (S.C.); gengshenggs@126.com (S.G.); zh8941@126.com (H.Z.); 2 Department of Food Science and Engineering, Jinan University, Guangzhou 510632, China

**Keywords:** flavonoid, *Ampelopsis grossedentata*, natural deep eutectic solvent, ultrasound-assisted extraction, antioxidant activity, antiproliferative activity

## Abstract

We performed ultrasound-assisted extraction coupled with natural deep eutectic solvents (NADES) to achieve the green and efficient preparation of flavonoid extract from *Ampelopsis grossedentata* leaves. We then evaluated its antioxidant and antiproliferative activities. A NADES consisting of choline chloride and glucose at a molar ratio of 4:1 with 20% water was determined to be the most suitable solvent. The optimal extraction conditions were: a liquid-to-solid ratio of 30 mL/g, an ultrasonication power of 490 W, and an ultrasonication time of 6.5 min. The actual flavonoid yield was 83.93%, which was close to the predicted yield. Further, 86.75% of the flavonoids were recovered by adding the same volume of phosphate buffer saline (100 mM, pH of 7.0) to the extract solution. Although the chemical antioxidant activities of the flavonoid extract were slightly inferior to those of dihydromyricetin, the flavonoid extract could still effectively inhibit the proliferation of human breast MDA-MB-231 cells by inducing cell apoptosis, retarding the cell cycle, changing the mitochondrial membrane potential and scavenging intracellular reactive oxygen species (ROS). The obtained results can provide a reference in the development of plant-derived functional foods.

## 1. Introduction

*Ampelopsis grossedentata* leaves have been used as a health tea in southern China for hundreds of years, as they possess antioxidant, anti-inflammatory, antihypertensive, antidiabetic, antibacterial, antiviral, anti-tumor, and other biological properties [1,2,3]. Artificial cultivation of *Ampelopsis grossedentata* has been achieved, and in 2013, the Chinese government approved *Ampelopsis grossedentata* as a new food resource [4]. *Ampelopsis grossedentata* leaves are rich in dihydromyricetin (DMY), myricetin, myricitrin, and other flavonoids, and the total flavonoid content can reach 30% [5,6]. Because flavonoids have poor solubility in water, flavonoid extraction using water has certain disadvantages such as high water and energy consumption and a difficult post-treatment process. Thus, extraction of flavonoids from *Ampelopsis grossedentata* leaves mainly uses traditional organic solvents such as methanol and ethanol [5,7]. However, these solvents are volatile and flammable. As a result, the development of a new green and efficient extraction technology is needed.

Considering the shortcomings of traditional organic solvents, a variety of new solvents, such as ionic liquids (ILs) and deep eutectic solvents (DESs), have been developed. However, ILs do have some disadvantages, such as complex synthesis, high production costs, potential toxicity, and low biodegradation rates; thus, they are not suitable for the practical applications of natural product extraction [8]. DESs are eutectic mixtures composed of hydrogen-bonded acceptors (HBAs) and hydrogen-bonded donors (HBDs) in specific proportions [9]. DESs overcome the shortcomings of ILs and have advantages such as flexible design ability, easy preparation, and high stability [9]. However, due to the toxicity of some DESs, their applications in the food industry have been limited [10]. Thus, a special type of DES, natural deep eutectic solvents (NADES), which are made entirely from food ingredients, have emerged. NADES have obvious advantages such as high safety, good biocompatibility, and excellent biodegradability [11]. They have also been successfully used for the extraction of polyphenols, lignin, polysaccharides, proteins, and other natural products [12,13,14]. 

Ultrasound-assisted extraction uses mechanical effects such as strong oscillation and cavitation to effectively infiltrate the solvent into the plant cell wall, which can significantly improve extraction efficiency [15]. Compared with traditional solvent extraction methods, it has the advantages of low energy consumption, short time and low equipment requirements. Therefore, NADES are usually coupled with ultrasound-assisted extraction. Although Wang et al. reported the extraction of DMY from *Ampelopsis grossedentata* leaves using β-cyclodextrin-based and ionic liquid-based ultrasonic-assisted extraction methods [16], as far as we know, there is no research on extracting DMY with ultrasound-assisted NADES extraction.

Choline chloride (CC) belongs to the vitamin B family and is generally recognized as safe as a dietary supplement by the United States Food and Drug Administration (FDA) [17]. In this study, NADES consisting of CC as the HBA and citric acid, glucose, and glycerol as the HBDs were constructed and coupled with an ultrasound-assisted extraction technique to extract the flavonoids from *Ampelopsis grossedentata* leaves. Flavonoid recovery was established based on the antisolvent method, and the antioxidant and antitumor activities of the obtained flavonoid extract were systematically evaluated.

## 2. Materials and Methods

### 2.1. Materials and Reagents

*Ampelopsis grossedentata* leaves (moisture content 9.42%) were purchased from Zhangjiajie, Hunan province, China and were crushed through a 200-mesh sieve. Dihydromyricetin (≥98%), CC (≥98%), citric acid (≥99.5%), glycerol (≥99%), and glucose (≥99%) were obtained from Aladdin (Shanghai, China). In addition, 2,2′-azobis (2-methylpropionamidine) dihydrochloride (ABAP), fluorescein sodium salt, Trolox, and 2′,7′-dichlorofluorescein diacetate (DCFH-DA) were purchased from Sigma-Aldrich LLC (Shanghai, China). Annexin V-FITC/PI apoptosis, 2′,7′-dichlorofluorescein diacetate (DCFH-DA), PI cell cycle, and JC-1 detection kits were obtained from Becton, Dickinson Company (Franklin Lakes, NJ, USA). Human hepatoma (HepG2), hepatocyte (LO-2), breast cancer (MDA-MB-231), and colorectal cancer (Caco-2) cell lines were obtained from the Cancer Institute of Sun Yat-Sen University (Guangzhou, China). Cell cultural media, penicillin, and streptomycin were purchased from Gibco Life Technologies (Grand Island, NY, USA). Fetal bovine serum (FBS) was obtained from Tianhang Biotech Co. Inc (Zhejiang, China). HPLC grade methanol was purchased from Fisher Chemical (Ottawa, ON, Canada). Ultrapure water was obtained from a Thermo Gen Pure ultraviolet (UV)/ultrafiltration water system (Waltham, MA, USA). All other chemicals were of analytical grade.

### 2.2. Preparation of the NADES

The CC-based NADES were prepared according to the methods described in a previous report [11]. CC was mixed with the HBDs (citric acid, glycerol, and glucose) at different molar ratios (Table 1). Then, the obtained mixture was stirred and heated at 80 °C until a homogeneous liquid formed. The cooled liquid was then sealed and stored for the following experiments.

### 2.3. Measurement of Total Flavonoid Content

First, 0.5 g of powdered *Ampelopsis grossedentata* leaves was dispersed in 200 mL of ethanol, refluxed at 80 °C for 6 h, and then filtered. The filtrate was diluted to 250 mL and collected as the sample solution. After the sample solution was properly diluted, its absorbance at 290 nm was determined using a PERSEE TU-1810PC UV spectrophotometer (Beijing, China). By comparing with the DMY standard curve, the flavonoid content in the leaves (%) was calculated, which was used as the total flavonoid content for the following extraction experiment.

### 2.4. Extraction Procedure

First, 0.5 g of powdered *Ampelopsis grossedentata* leaves was mixed with NADES at the specified liquid-to-solid ratio in a beaker. The mixture was ultrasonically treated at 30 °C with the selected powers for the designated times using a KQ-5200DE ultrasonicator (Kun Shan Ultrasonic Instruments Co., Ltd., Kunshan, China) and centrifuged at 7000 rpm for 10 min. The flavonoid concentration in the supernatant was determined according to Section 2.3. The corresponding flavonoid yield (Y) was calculated based on the following Equation (1):

(1) $$Y=\frac{C_{1}}{C_{0}}\cdot 100\%$$

where C_1_ and C_0_ are the flavonoid quantity extracted and the flavonoid quantity in the raw materials, respectively.

### 2.5. Optimization of Ultrasound-Assisted NADES Extraction

The optimization of ultrasound-assisted NADES extraction was carried out in three steps. First, the effects of the CC-based NADES listed in Table 1, with and without the addition of water (10–30 vol%), were evaluated on the flavonoid yield, with ethanol and water as the reference solvents to screen for the optimal NADES. Then, the significant factors that influence ultrasound-assisted extraction were investigated using a single factor experiment (ultrasonication time, 2.5–30 min; ultrasonication power, 280–700 W; liquid-to-solid ratio, 10:1–60:1). Finally, the extraction conditions were optimized with the flavonoid yield (Y) as the response, using the central composite design (CCD) of the response surface methodology (RSM). The three coded variables (liquid-to-solid ratio (X_1_), ultrasonication power (X_2_), and ultrasonication time (X_3_)) and their levels (−1, 0, 1) are listed in Table 2. The obtained results were fitted to the quadratic polynomial model [18], the equation (2) is as follows:

(2) $$Y=\beta_{0}+\sum \beta_{i}\cdot X_{i}++\sum \beta_{\mathrm{ij}}\cdot X_{i}\cdot X_{j}+\sum \beta_{ii}\cdot X_{i}^{2}$$

where β_0_ is a constant, and β_i_, β_ij_, and β_ii_ denote the linear, interaction, and quadratic regression coefficients, respective.

By using Design-Expert 7.0 software (Stat-Ease Inc., Minneapolis, MN, USA), we determined the optimal conditions, and their adequacy was verified by the additional experiments.

### 2.6. Recovery of the Flavonoid Extract

The flavonoids in the NADES were recovered using the anti-solvent method. The extract solution obtained under the optimal extraction conditions was mixed with phosphate buffer saline (PBS) at different amounts and pH values, magnetically stirred for 1 h at room temperature, and then filtered. The precipitate was vacuum dried at 60 °C, weighed to calculate the flavonoid recovery value, and collected as the flavonoid extract. The flavonoid content was measured according to Section 2.3. DMY content in the extract was determined using an Agilent 1260 HPLC system (Santa Clara, CA, USA), which was equipped with an Agilent ZORBAX SB-C18 column (150 mm × 4.6 mm, 5 μm particle size) at 30 °C, and the detection wavelength was 290 nm. The injection volume was 10 μL. The mobile phase consisted of methanol and water (8:2) with a flow rate of 1 mL/min. Quantification of DMY was based on the external standard method using Agilent OpenLAB ChemStation software (Santa Clara, CA, USA) [19].

### 2.7. Measurement of the Oxygen Radical Absorbance Capacity (ORAC)

The oxygen radical absorbance capacity (ORAC) assay was carried out according to the methods reported by Chen et al. [20]. Different concentrations of the sample (flavonoid extract or DMY) and Trolox solution were injected into the wells of a 96-well black plate with a clear bottom polystyrene microplate and incubated at 37 °C for 10 min. Fluorescein sodium salt working solution was added to each well, followed by incubation for another 20 min at 37 °C. Then, ABAP working solution was added to the test wells. Fluorescence was measured at an excitation wavelength of 485 nm and an emission wavelength of 535 nm for 35 cycles every 4 min using a Fluoroskan Ascent fluorescence spectrophotometer (Tecan M200 PRO, Switzerland). The ORAC results were expressed as micromoles of Trolox equivalent (TE) per milligram of sample.

### 2.8. Measurement of Hydrophilic Peroxyl Radical Scavenging Capacity (Hydro-PSC)

Hydro-PSC measurement was performed according to a previous report [21]. The sample (flavonoid extract or DMY) and vitamin C solutions at different concentrations were diluted using pH 7.4 PBS solution and injected into a 96-well black plate with a clear bottom polystyrene microplate. Afterwards, DCFH-DA was hydrolyzed by KOH for 5 min in the dark and diluted with PBS to 8 mL of the total volume of the working solution. The ABAP working solution was prepared using fresh PBS. Then, the DCFH-DA and ABAP working solutions were successively injected into each well. Fluorescence changes were monitored at 37 °C using a fluorescence spectrophotometer. The Hydro-PSC results were expressed as micromoles of vitamin C equivalent (VCE) per gram of sample.

### 2.9. Cell Culture

The human hepatoma (HepG2), hepatocyte (LO-2), breast cancer (MDA-MB-231), and colorectal cancer (Caco-2) cell lines were fostered in a DMEM containing 10% FBS, 50 μg/mL streptomycin, and 50 units/mL penicillin. The cells were incubated at 37 °C with 5% CO_2_, which was used for facilitating exponential growth.

### 2.10. Cell Viability Assay

The antiproliferative activities of the samples (flavonoid extract and DMY) on the selected cells were evaluated by MTT. Cells were seeded on a 96-well white microplate at a density of 2.5 × 10^4^ cells/ (100 μL × well). Incubation was performed for 12 h to allow the cells to attach, and then, the growth medium was suctioned out and the cells were lightly washed with PBS. Afterward, sample working solutions at different concentrations were added to the wells, and the medium without the sample solution was set as the control group. Incubation was performed for 48 h, and then, the cells were washed with PBS and stained with MTT working solution. The stained cells were flooded with dimethyl sulfoxide (DMSO), the plate was shaken for 10 min at room temperature, and the samples were analyzed using a multifunctional spectrophotometer at 570 nm.

### 2.11. Cell Apoptosis Assay

The effect of flavonoid extract on cell apoptosis of the MDA-MB-231 cells was assessed using an annexin-V-FITC/PI apoptosis detection kit. The MDA-MB-231 cells were incubated with the flavonoid extract working solutions (0, 50, 75, and 100 μg/mL) for 48 h, and the gathered cells were stained according to the detection kit methods. Then, fluorescence intensity was monitored using a FACSCanto cell analyzer (Becton, Dickinson & Co., Franklin Lakes, NJ, USA). For each sample, 10,000 events were recorded by flow cytometry. The data were collected and analyzed by CellQuest research software.

### 2.12. Cell Cycle Assay

The cell cycle of MDA-MB-231 was detected using a PI staining detection kit. The MDA-MB-231 cells were treated with different concentrations of flavonoid extract for 48 h (0, 50, 75, and 100 μg/mL), and then, the treated cells were collected and stained with a PI staining detection kit(Becton, Dickinson and Company, Franklin Lakes, NJ, USA). The fluorescence intensities of the stained cells were monitored using a FACSCanto cell analyzer. For each sample, 10,000 events were recorded by flow cytometry, and analyzed by CellQuest research software.

### 2.13. Measurement of Mitochondrial Membrane Potential

The effect of flavonoid extract on the mitochondrial membrane potential of the MDA-MB-231 cells was investigated by flow cytometry according to a detection kit. The cells were treated with flavonoid extract working solutions at different concentrations (0 and 50 μg/mL), collected, and then stained with a JC-1 detection kit (Becton, Dickinson and Company, Franklin Lakes, NJ, USA). Fluorescence was monitored with a FACSCanto cell analyzer ( Becton, Dickinson and Company, Franklin Lakes, NJ, USA).

### 2.14. Measurement of Reactive Oxygen Species (ROS)

The ROS scavenging activity of the flavonoid extract was assessed using a detection kit. The MDA-MB-231 cells were seeded on a 96-well black plate with a clear bottom polystyrene microplate at a density of 2.5 × 10^4^ cells/(100 μL × well). Incubation was performed for 12 h to allow the cells to attach, and then, the growth medium was suctioned out and the cells were lightly washed with PBS. Then, the cells were treated with the flavonoid extract at different concentrations for 24 h (50, 100, 200, 300, and 400 μg/mL). The treated cells were stained for 30 min according to a ROS staining detection kit. Then, the fluorescence intensity of the stained cells was monitored 20 times every 2 min using a Fluoroskan Ascent fluorescence spectrophotometer (Tecan M200 PRO, Switzerland).

### 2.15. Statistical Analysis

All measurements were performed in triplicate and expressed as mean ± standard deviation. Statistical comparisons were carried out using SPSS software package based on the Duncan test with a confidence level of 95%.

## 3. Results and Discussion

### 3.1. Selection of the NADES

**Figure 1:**
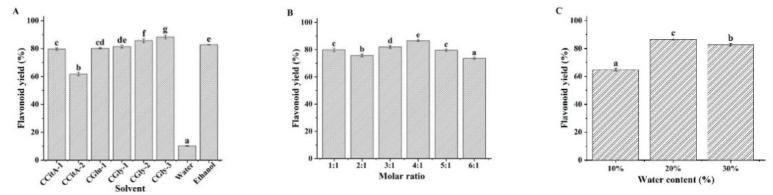
Effects of composition (**A**), molar ratio (**B**), and water content (**C**) of the NADES on flavonoid yield. (Bars with different letters differ significantly at *p* < 0.05).

NADES are composed of hydrogen bond donors and acceptors at a certain molar ratio, and their composition not only affects their physicochemical properties (density, viscosity, and polarity) but also their solubility and ability to dissolve the target component [22]. In this study, six CC-based NADES were prepared (Table 1), and their flavonoid extraction efficiencies were compared with water and ethanol as the reference solvents. As shown in Figure 1A, the flavonoid yields of all the NADES were higher than those of water. Besides CCitA-2, the flavonoid yields of the other NADES were similar to those of ethanol, which is the most commonly used organic solvent for flavonoid extraction. Compared to citric acid and glycerol, glucose may be more suitable for practical applications in foods. Therefore, the NADES constructed with CC and glucose (CGlu) was selected for further evaluation. The HBA/HBD molar ratio of NADES will directly affect their stability and ability to dissolve the target component. To test this, the flavonoid yields of CGlu solvents developed with molar ratios ranging from 1:1 to 6:1 were evaluated. As shown in Figure 1B, their flavonoid yields were all higher than 70%, and the CGlu solvent developed at a molar ratio of 4:1 exhibited the highest yield. The viscosities of the NADES were much higher than those of water, ethanol, and other traditional solvents, which greatly hindered mass transfer during the extraction process [23]. Although ultrasound-assisted extraction can partially overcome this problem, the effect is not ideal. Reports have shown that the addition of a small amount of water can significantly reduce NADES viscosity and increase their solubilizing capacity [24]. The optimal water content was dependent on the NADES type and the extracted target component. In this case, we investigated the effect of water content on the flavonoid yield of CGlu developed at a molar ratio of 4:1 (Figure 1C). The appropriate addition of water could enhance the flavonoid extraction efficiency, and the highest yield was obtained with a water content of 20%. The previous report also indicated the CGlu with a water content of 25% could extract flavonoids from citrus peel waste [25]. However, excessive dilution could disrupt the hydrogen bonds between the HBDs and HBAs in the NADES, which could deteriorate flavonoid extraction efficiency. Based on the above results, the CGlu solvent developed at a molar ratio of 4:1 with 20% water (CGlu-W20) was selected as the suitable solvent for the following flavonoid extraction experiments.

### 3.2. Single-Factor Experiment of Extraction

**Figure 2:**
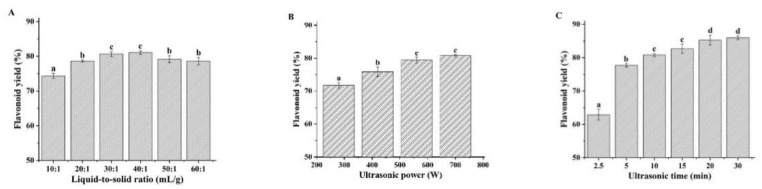
Effects of liquid-to-solid ratio (**A**), ultrasonication power (**B**), and ultrasonication time (**C**) on ultrasound-assisted extraction of flavonoids. (Bars with different letters differ significantly at *p* < 0.05).

When the extraction solvent has been determined, the operation parameters such as ultrasonication power, ultrasonication time, and the liquid-to-solid ratio have significant effects on the extraction efficiency [26]. In this study, we evaluated the effects of these parameters on the flavonoid yield with CGlu-W20 as the extraction solvent using a single factor experiment (Figure 2), which provided a reference for the RSM experiment. Typically, an increase in the liquid-to-solid ratio can reduce the concentration of the target component in the solution, which is conducive to promoting diffusion from plant tissues. However, if the liquid-to-solid ratio is too large, it will cause difficulties in subsequent treatments, resulting in a lower yield. In this experiment, when the ultrasonication power and time were fixed at 420 W and 5 min, respectively, the flavonoid yield increased with increasing liquid-to-solid ratio and decreased slightly when the liquid-to-solid ratio exceeded 30 (Figure 2A). To reduce the amount of solvent and the burden of subsequent treatments, the test range for the liquid-to-solid ratio in the RSM experiment was determined to be 10–30. As shown in Figure 2B, when the ultrasonication time was 5 min and the liquid-to-solid ratio was 20, the flavonoid yield increased with increasing ultrasonication power; however, it slowed down when it exceeded 560 W, which was attributed to the increase in ultrasonication power, which gradually strengthened the cavitation effect and exacerbated the breakdown of plant tissue. However, when the ultrasonication power exceeded a specific value, a saturation effect would occur. Therefore, to reduce energy consumption, the range of variation in ultrasonication power in the RSM experiment was set to 280–560 W. Figure 2C shows the changes in flavonoid yield with ultrasonication time when the ultrasonication power was set to 420 W and the liquid-to-solid ratio was 20. In the initial stage, with extended extraction, the flavonoid yield increased rapidly. However, after 15 min, it increased slowly, possibly because the plant cell wall was completely broken down; thus, the flavonoid yield became stable. To save energy and reduce ultrasonication time, the factor range of ultrasonication time in the RSM experiment was set to 5–15 min. It was also observed that ultrasonic treatment led to a slight increase in the temperature of NADES, which was conducive to reducing the viscosity of NADES and improving the mass-transfer rate in the extraction process [27,28,29].

### 3.3. Extraction Optimization 

**Figure 3:**
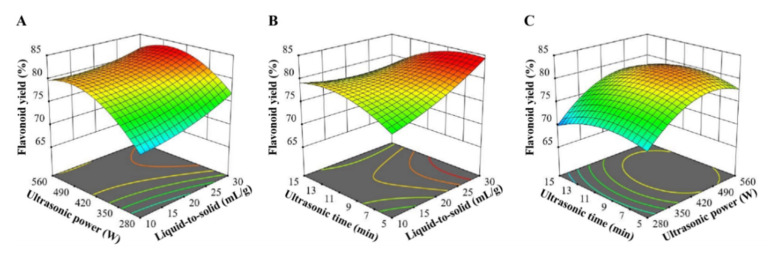
Response surface plots for ultrasound-assisted extraction of flavonoids from *Ampelopsis grossedentata* leaves; the interaction between ultrasonic power and liquid-to-solid ratio (**A**), ultrasonic time and liquid-to-solid ratio (**B**), ultrasonic time and ultrasonic power (**C**).

Based on the single-factor experimental results, the RSM experiment was carried out to optimize the ultrasound-assisted extraction of flavonoids in *Ampelopsis grossedentata* leaves with CGlu-W20 as the solvent. RSM, a statistical method to solve multivariable problems, utilizes reasonable experimental design and obtains certain data through experiments, uses multiple quadratic regression equation to fit the functional relationship between factors and response values, and seeks the optimal process parameters by analyzing the regression equation, which has been effectively applied to optimize the process parameters in the extraction and modification of bioactive compounds [30,31]. The RSM experimental design and results are presented in Table 2. The corresponding second-order polynomial equation with independent variables (liquid-to-solid ratio (X_1_), ultrasonication power (X_2_), and ultrasonication time (X_3_)) and response variable (flavonoid yield (Y)) was also obtained (Table 2). The ANOVA results suggested that the model was highly significant (*p* < 0.0001). Both the high determination coefficient (R^2^ = 0.9872) and the non-significant lack of fit (*p* = 0.1534) indicated a good fit of this model, which was adequate for reasonable predictions within the experimental range. The linear terms (X_1_, X_2_, and X_3_), interaction terms (X_1_X_2_ and X_1_X_3_), and quadratic terms (X_2_^2^ and X_3_^2^) had a significant influence on flavonoid extraction (*p* < 0.05), and their effects are visually demonstrated in Figure 3. These three-dimensional plots had similar patterns, in which the liquid-to-solid ratio had a positive effect on the flavonoid yield; however, the flavonoid yield did not simply rise with increasing ultrasonication time or power. This may be attributed to the instability of flavonoids under severe treatment conditions [32]. When the variation ranges of the influencing factors were set within the experimental range, the optimal extraction conditions could be obtained by using the second-order polynomial equation (Table 2) as follows: a liquid-solid ratio of 30 mL/g, an ultrasonication power of 458.12 W, and an ultrasonication time of 6.48 min. Considering actual operations, verification tests were carried out under the following conditions: a liquid-solid ratio of 30 mL/g, an ultrasonication power of 490 W, and an ultrasonication time of 6.5 min. The actual flavonoid yield was 83.93 ± 0.54%, which was consistent with the predicted yield of 84.85%, confirming the reliability of the model. The established ultrasound-assisted NADES extraction of flavonoids takes only 6.5 min to extract more than 80% of flavonoids from *Ampelopsis grossedentata* leaves, which is superior to the traditional solvent extraction method [7,30] and close to the ionic liquid-based ultrasonic-assisted extraction method reported by Wang et al. [16].

### 3.4. Optimization of Flavonoid Recovery

**Figure 4:**
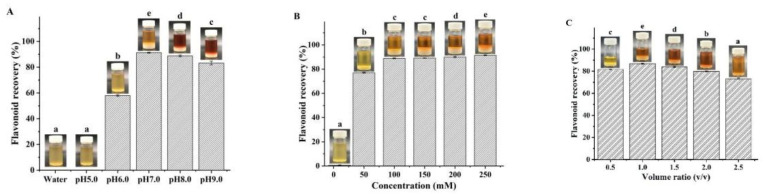
Effects of pH (**A**), concentration (**B**), and PBS addition amount (**C**) on flavonoid recovery. (Bars with different letters differ significantly at *p* < 0.05).

Due to their negligible vapor pressure, flavonoid recovery from NADES is extremely difficult. In previous reports, several methods have been attempted, including the anti-solvent, macroporous resin adsorption, and solid-phase extraction methods [17,33,34]. In this study, we first attempted to recover flavonoids with water as the anti-solvent. However, contrary to expectations, regardless of how much water was added, the flavonoids failed to precipitate out, which was attributed to the strong hydrogen bonds between the NADES and the flavonoids [11]. Thus, we used PBS as the anti-solvent to precipitate the flavonoids, since PBS could not only destroy the hydrogen bonds between the HBA and HBD of NADES, but also destroy the hydrogen bonds between NADES and flavonoids. The effects of concentration, pH, and PBS addition amount on flavonoid recovery are shown in Figure 4. We observed the highest flavonoid recovery at a pH of 7.0 (Figure 4A), when the PBS concentration was 200 mM and the addition volume ratio was fixed at 1. When the pH was 7.0 and the addition volume ratio was 1.0, PBS at a concentration of 100 mM exhibited the highest separation effect (Figure 4B). Therefore, 100 mM of PBS with a pH of 7.0 was determined to be a suitable anti-solvent for flavonoid recovery. As shown in Figure 4C, when the optimal addition volume ratio was 1.0, the resulting flavonoid recovery content was 86.75 ± 0.32%. Flavonoid content in the obtained extract was 75.24 ± 0.23%, while DMY content was 69.55 ± 2.38%, which was used for the subsequent antioxidant and antiproliferative assays. To the best of our knowledge, this is the first report of using PBS to recover flavonoids from NADES.

### 3.5. Chemical Antioxidant Activity

**Figure 5:**
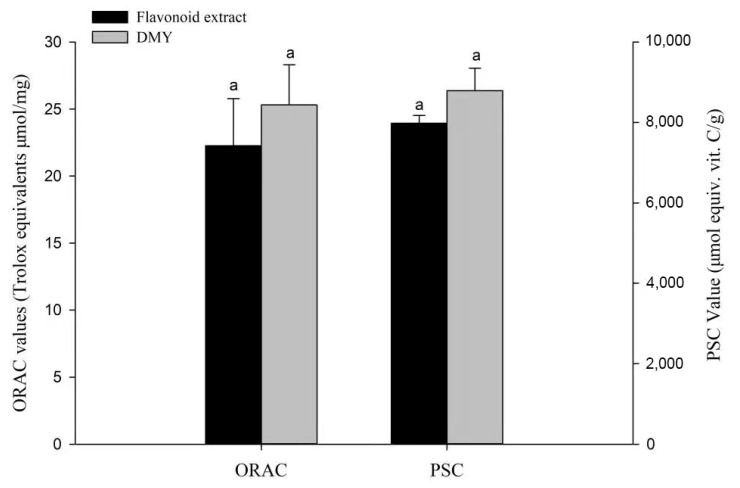
Chemical antioxidant activities of the flavonoid extract and DMY. Bars with letters differ significantly at *p* < 0.05.

Many phytochemicals, such as phenolic acids and flavonoids, contribute to the antioxidant characteristics of fruits and vegetables, and their potential nutritional value and safety have been widely assessed [35]. The previous report reported the superior antioxidant activity of the extract of *Ampelopsis grossedentata* leaves [36]. Due to the complex reaction mechanisms of phytochemicals, it is necessary to study their antioxidant activity using accurate and multiple evaluation methods. Considering this, we used PSC and ORAC chemical antioxidant assays in this study. As shown in Figure 5, the antioxidant performance of DMY was slightly superior to that of the flavonoid extract, which coincided with a previous report that showed that DMY possessed higher antioxidant activity than the water extract of *Ampelopsis grossedentata* leaves [19]. 

### 3.6. Antiproliferative Activity

**Figure 6:**
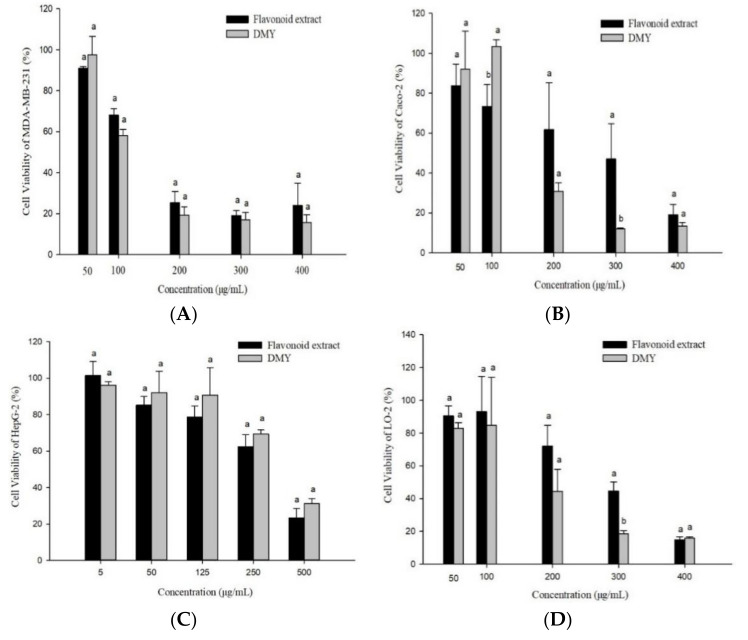
Anti-proliferative activities of the flavonoid extract and DMY against MDA-MB-231 (**A**), Caco-2 (**B**), HepG2 (**C**), and LO-2 (**D**) cells. (Bars with different letters differ significantly at *p* < 0.05).

Cancer is one of three major diseases that threaten human life and health. It has the highest mortality rate, besides heart disease and infectious diseases, and accounts for one-fourth of all deaths every year [37]. Anti-cancer drugs include synthetic drugs and natural plant extracts. Plant extracts have certain advantages such as few side effects, unique effects, and low research costs. They have also been widely used as antineoplastic drugs or adjuvant drugs [38]. Their antiproliferative activities can be preliminarily evaluated by the cell viability and selectivity index [27,39]. In this study, we investigated the antiproliferative activity of flavonoid extract against MDA-MB-231, Caco-2, and HepG2 cancer cells with LO-2 cells (normal hepatocytes) as the control (Figure 6). The flavonoid extract had the strongest inhibitory activity toward MDA-MB-231, followed by Caco-2 and HepG2. DMY showed the strongest inhibitory activity toward MDA-MB-231, followed by Caco-2 and HepG2. DMY also had higher inhibitory activity toward MDA-MB-231 and Caco-2 cells than the flavonoid extract. However, the flavonoid extract showed stronger inhibitory activity toward the HepG2 cells than DMY. Furthermore, the flavonoid extract showed weaker inhibitory activity toward the LO-2 cells than DMY. Overall, the flavonoid extract was effective across the three cancer cell lines and showed the most potent inhibition activity toward the MDA-MB-231 cells. Therefore, in the follow-up study, we investigated the antiproliferative mechanism of the flavonoid extract against MDA-MB-231 cells.

### 3.7. Cell Apoptosis

**Figure 7:**
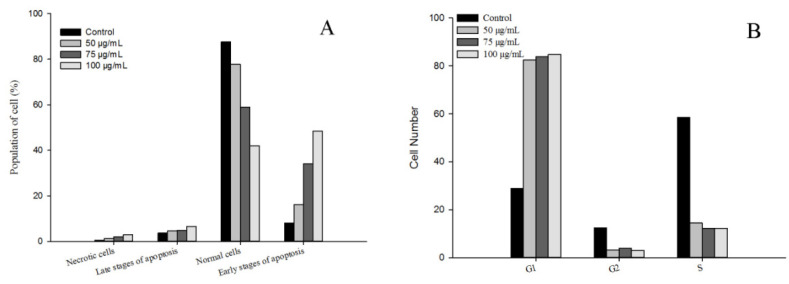
Effect of flavonoid extract on cell apoptosis (**A**) and the cell cycle (**B**).

**Figure 8:**
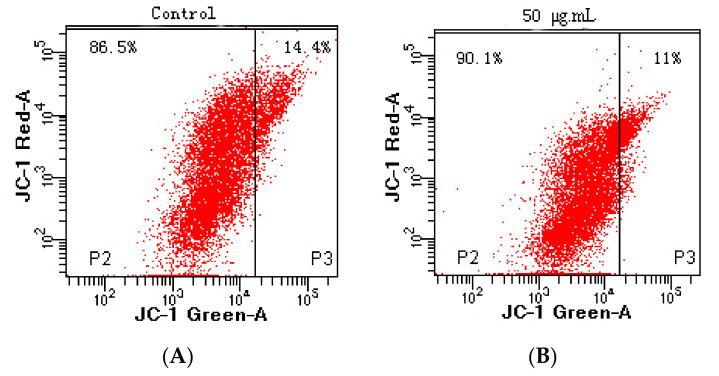
Effect of flavonoid extract on the mitochondrial membrane potential. ((**A**), 0 μg/mL; (**B**), 50 μg/mL).

Apoptosis, also known as programmed cell death, refers to the so-called suicidal behavior of cells, where they die by controlling internal genes under the action of certain physiological or pathological factors [40]. Apoptosis is a normal function in multicellular organisms, and this process can remove aging, pathological, and abnormal cells to maintain the best physiological function [41]. Numerous studies have shown that anti-cancer drugs initiate this process mainly by inducing tumor cell apoptosis, which is characterized by DNA fragmentation, cell cycle arrest, and mitochondrial membrane damage [42]. In this study, the effects of flavonoid extracts at different concentrations (0, 50, 75, and 100 μg/mL) on cell apoptosis of MDA-MB-231 cells were evaluated (Figure 7A). We observed that with increasing flavonoid extract concentration, the proportion of normal cells decreased rapidly, the proportion of cells in the early apoptotic stage increased significantly, and the proportion of cells in the late apoptotic stage declined slightly, suggesting that the apoptosis-induced activity of the flavonoid extract was in the early stage of apoptosis. Similarly, the flavonoid extract caused changes in the cell cycle of the MDA-MB-231 cells. The ratio of G1 phase arrest in the MDA-MB-231 cells increased from 28.90% (blank) to 84.70% (100 μg/mL flavonoid extract), indicating that the flavonoid extract inhibited DNA synthesis and arrested the proliferation of MDA-MB-231 cells in the G1 phase. Mitochondrial damage caused by cell apoptosis has been generally demonstrated by an increase in membrane permeability and a decrease in membrane potential [43]. JC-1 is a mitochondrial-targeted fluorescent probe, where normal cells exhibit red fluorescence, while apoptotic cells exhibit green fluorescence. As shown in Figure 8, P2 and P3 represent green and red fluorescence, respectively. Treatment with flavonoid extract led to a fluorescence shift from red to green, which confirmed that the flavonoid extract caused mitochondrial dysfunction. In summary, the flavonoid extract could induce cell apoptosis by retarding the cell cycle and changing the mitochondrial membrane potential.

### 3.8. ROS Scavenging Activity

**Figure 9:**
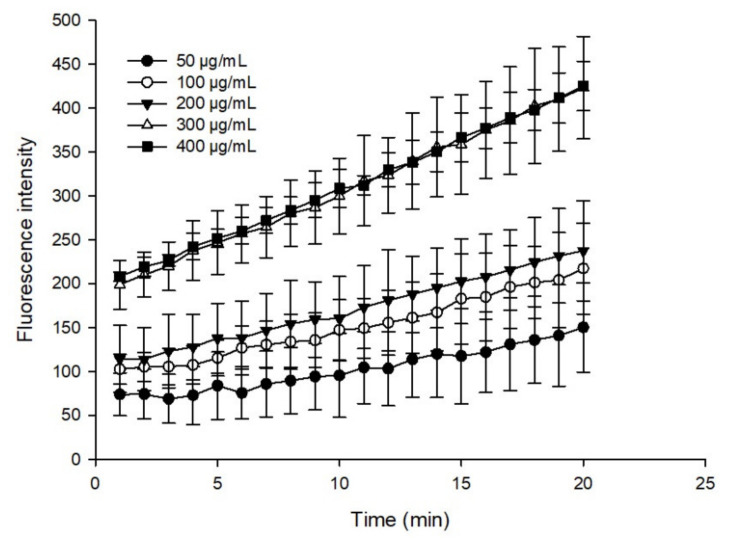
ROS scavenging performance of the flavonoid extract.

Oxidative damage caused by ROS is an important factor that drives the occurrence and development of cancer [44]. Growing evidence has demonstrated that cancer cells appear due to increased intrinsic ROS stress [45]. Generally, the human body is equipped with various antioxidants, which can be divided into enzymatic and nonenzymatic. Nonenzymatic antioxidants include phenolics, flavonoids, β-carotene, ascorbic acid, and α-tocopherol [46]. These compounds have important potential roles in anticancer strategies, such as modulating ROS content in cancer cells. In this study, the intracellular ROS scavenging activity of the flavonoid extract was dynamically monitored using DCFH-DA dye. Figure 9 shows the sample fluorescence intensity results after deducting the blank, where the higher fluorescence intensity corresponded to a weaker fluorescence scavenging ability. The flavonoid extract could effectively remove ROS in a dosage-dependent manner, and the scavenging effect of the flavonoid extract was similar, at 400 and 500 μg/mL, which was possibly due to cell inhibition at higher concentrations. These results indicated that preincubation with the flavonoid extract reduced oxidative stress.

## 4. Conclusions

In this study, we achieved the efficient and green extraction of flavonoids from *Ampelopsis grossedentata* leaves, using CGlu-W20 as the solvent under ultrasound-assisted extraction, with the following conditions: a liquid-to-solid ratio of 30 mL/g, an ultrasonication power of 490 W, and an ultrasonication time of 6.5 min. The flavonoids in the NADES extract were recovered when PBS (100 mM and pH of 7.0) was used as the anti-solvent with an addition volume ratio of 1.0. The obtained flavonoid extract exhibited excellent ORAC and PSC antioxidant activity and could inhibit the proliferation of human breast MDA-MB-231 cells by inducing cell apoptosis, retarding the cell cycle, changing the mitochondrial membrane potential, and scavenging intracellular ROS. The obtained results can provide a reference in the development of plant-derived functional foods.

## Figures and Tables

**Table 1:** List of the NADES employed in this study.

Abbreviation	HBA	HBD	Mole Ratio
CCitA-1	Choline chloride	Citric acid	1:1
CCitA-2	Choline chloride	Citric acid	1:2
CGlu-1	Choline chloride	Glucose	1:1
CGly-1	Choline chloride	Glycerol	1:1
CGly-2	Choline chloride	Glycerol	1:2
CGly-3	Choline chloride	Glycerol	1:3

**Table 2:** Experimental design and RSM results.

RSM Experiment					ANOVA					
Run	X_1_ (mL/g)	X_2_ (W)	X_3_ (min)	Y (%)	Source	Sum of Squares	df	Mean Square	F-Value	*p*-Value
1	20 (0)	280 (−1)	10 (0)	72.57 ± 0.97	Model	315.91	9	35.10	59.92	<0.0001
2	10 (−1)	420 (0)	10 (0)	79.55 ± 0.36	X_1_	37.25	1	37.25	63.58	<0.0001
3	20 (0)	420 (0)	15 (1)	78.34 ± 1.05	X_2_	110.22	1	110.22	188.15	<0.0001
4	10 (−1)	560 (1)	15 (1)	79.81 ± 0.36	X_3_	4.52	1	4.52	7.71	0.0274
5	20 (0)	420 (0)	10 (0)	80.90 ± 0.44	X_1_X_2_	6.18	1	6.18	10.55	0.0141
6	20 (0)	420 (0)	10 (0)	80.20 ± 0.87	X_1_X_3_	54.03	1	54.03	92.23	<0.0001
7	30 (1)	560 (1)	15 (1)	75.95 ± 0.40	X_2_X_3_	1.13	1	1.13	1.93	0.2070
8	20 (0)	560 (1)	10 (0)	80.20 ± 1.06	X_1_^2^	2.70	1	2.70	4.60	0.0690
9	10 (−1)	280 (−1)	5 (−1)	68.24 ± 0.18	X_2_^2^	43.01	1	43.01	73.42	<0.0001
10	30 (1)	560 (1)	5 (−1)	82.98 ± 0.94	X_3_^2^	13.52	1	13.52	23.08	0.0020
11	30 (1)	420 (0)	10 (0)	83.24 ± 0.69	Residual	4.10	7	0.59		
12	10 (−1)	560 (1)	5 (−1)	74.83 ± 1.36	Lack of fit	3.84	5	0.77	5.81	0.1534
13	20 (0)	420 (0)	10 (0)	80.38 ± 1.22	Credibility analysis of the regression equations					
14	20 (0)	420 (0)	5 (−1)	77.95 ± 0.92	Std. dev.	0.7654	R-squared	0.9872	Adeq. precision	27.0219
15	10 (−1)	280 (−1)	15 (1)	70.10 ± 0.38	Mean	77.35	Adj. R-squared	0.9707		
16	30 (1)	280 (−1)	5 (−1)	78.29 ± 1.43	Second-order polynomial equation					
17	30 (1)	280 (−1)	15 (1)	71.37 ± 0.80	Y = 80.44 + 1.93 ×X_1_ + 3.32 × X_2_ − 0.67 × X_3_ − 0.88 × X_1_ × X_2_ − 2.60 × X_1_ × X_3_ + 0.38 × X_2_ × X_3_ + 1.00 × X_1_^2^ − 4.01 × X_2_^2^ − 2.25 × X_3_^2^					

X_1_, liquid-to-solid ratio; X_2_, ultrasonication power; X_3_, ultrasonication time; Y, flavonoid yield.

## Data Availability

The data that support the findings of this study are available on request from the corresponding author. The data are not publicly available due to privacy or ethical restrictions.
